# Soy Protein Isolate-Phosphatidylcholine Nanoemulsions Prepared Using High-Pressure Homogenization

**DOI:** 10.3390/nano8050307

**Published:** 2018-05-07

**Authors:** Yang Li, Chang-Ling Wu, Jun Liu, Ying Zhu, Xiao-Yuan Zhang, Lian-Zhou Jiang, Bao-Kun Qi, Xiao-Nan Zhang, Zhong-Jiang Wang, Fei Teng

**Affiliations:** 1 College of Food Science, Northeast Agricultural University, Harbin 150030, China; liyang@neau.edu.cn (Y.L.); wuchangling@neau.edu.cn (C.-L.W.); zhuying@neau.edu.cn (Y.Z.); zhangxiaoyuan@neau.edu.cn (X.-Y.Z.); jianglianzhou@neau.edu.cn (L.-Z.J.); qibaokun@neau.edu.cn (B.-K.Q.); xiaonanzhang@neau.edu.cn (X.-N.Z.); 2 Shan Dong Yuwang Ecological Food Industry, Yucheng 251200, China; liujun@yuwangcn.com

**Keywords:** soy protein isolate, phosphatidylcholine, nanoemulsions, high-pressure homogenization

## Abstract

The nanoemulsions of soy protein isolate-phosphatidylcholine (SPI-PC) with different emulsion conditions were studied. Homogenization pressure and homogenization cycle times were varied, along with SPI and PC concentration. Evaluations included turbidity, particle size, ζ-potential, particle distribution index, and turbiscan stability index (TSI). The nanoemulsions had the best stability when SPI was at 1.5%, PC was at 0.22%, the homogenization pressure was 100 MPa and homogenization was performed 4 times. The average particle size of the SPI-PC nanoemulsions was 217 nm, the TSI was 3.02 and the emulsification yield was 93.4% of nanoemulsions.

## 1. Introduction

Nanoemulsions are emulsions where the droplets are 100 nm to 500 nm [1]. The advantages of nanoemulsions are high optical clarity, good physical stability against gravitational separation and droplet aggregation, and enhanced bioavailability of encapsulated substances, which make them suitable for food applications [2], such as for food processing, packaging, safety, nutrition and nutraceuticals [3]. Nanoemulsions have been mainly produced by homogenization, either using high-pressure homogenization or microfluidization. Microfluidization uses high pressure to force the fluid through microchannels that have a specific configuration, emulsifying the fluid by the combined effects of cavitation, shear and impact, thus resulting in excellent emulsifying efficiency [4]. However, the microfluidization method requires high energy and dedicated equipment, which leads to a high production cost [5]. High-pressure homogenization is one of the most efficient ways to make nanoemulsions that are uniform, and which have strong stability and high preparation efficiency. Studies by Molina et al. (2001) have shown that high-pressure homogenization can improve the emulsifying activity of soy proteins but, in most cases, it does not improve emulsifying stability [6]. Tan and Nakajima (2005) successfully prepared β-carotene nanoemulsions using high-pressure homogenization with a narrow size distribution of the β-carotene particles [7].

The surfactant is important for preparing emulsions. The most common proteins used in emulsion stabilization are dairy-based proteins such as casein and whey proteins. However, plant proteins and phosphatidylcholine may be used, and nanoemulsions as delivery systems have good emulsifying properties, binding capacity for hydrophobic bioactive compounds, and gelation properties. Plant proteins (e.g., proteins from soy, pea, lentil and canola) are less understood as emulsifiers compared to dairy proteins [2,8]. Soybean protein-lecithin complex shows better emulsifying activity with thermal treatment [9]. Henry et al. (2010) showed that phospholipids or food-grade proteins could be the sole interfacial species, because of the droplet size being dependent on both the breakup and coalescence of droplets at emulsifier concentrations below 1%, while only being dependent on the droplet breaking up at higher concentrations [10]. According to several authors, phospholipids may either displace proteins from the interface or can interact with the proteins either in solution or on the surface, and lecithin addition enhances the heat-stability of whey protein-stabilized O/W emulsions [11,12]. Kasinos et al. [13] reported that the heat stability of solutions and emulsions containing whey protein was greatly improved through the addition of sunflower lecithin. Our previous studies firstly discovered that the hydrophobic group at the sn-2 position of phosphatidylcholine (PC) is the main component of lecithin, and that it is used to determine the emulsifying activity of PC [14]. Then, we found that the ultrasonic treatment increased the Emulsifying Activity Index (EAI) and Emulsion Stability Index (ESI) of SPI-lecithin stabilized emulsion, but the continuous increases of ultrasonic power and duration had negative impacts on their emulsion stability [15]. Meanwhile, the effect of high-pressure homogenization on the emulsion properties and stability of SPI-phosphatidylcholine emulsions has not yet been systematically studied. The regulation and selection of suitable high-pressure homogenous preparation parameters have significant effects on the stability and function of the nanoemulsion. Therefore, this work aims at evaluating the effects of high-pressure homogenization on the properties of nanoemulsions prepared when stabilized by SPI and phosphatidylcholine, while SPI and PC were used as the emulsification phase, and sunflower seed oil was used as the oil phase. The interfacial structure and micro-morphology characteristics of high-pressure homogenous nanoemulsions preparations were observed and analyzed by Raman imaging technology to provide the theoretical basis and data support for nanoemulsions preparation and quality control. The results of this work would be useful in providing a novel and improved method of producing soybean protein-stabilized nanoemulsions.

## 2. Materials and Methods

### 2.1. Experimental Materials

Soybeans were obtained from Gao Tang Co., Ltd., Liao Cheng, Shandong Province, China. PC, with a content of acetone-insoluble material >95%, was obtained from Sigma-Aldrich (St. Louis, MO, USA). Sunflower oil (COFCO Co. Ltd., Harbin, Heilongjiang Province, China) was purchased from a local supermarket. All other chemicals were of analytical reagent grade was used throughout.

### 2.2. Preparation of Nanoemulsions

SPI were prepared according to the method of Niu, Xia, Wang, Kong and Liu (2018) [16]. SPI (0.5%, 1.0%, 1.5%, 2.0%, 2.5%, 3.0%) was mixed with PC (0.10%, 0.17%, 0.20%, 0.22%, 0.25%) with varying ratios before being dispersed in 0.05 M sodium phosphate buffer, pH 7.4, followed by mixing at room temperature (22 °C to 25 °C) for 30 min using magnetic stirring. β-carotene (≥97.0%, 0.01 g, *w*/*w*), (COFCO Co. Ltd., Harbin, Heilongjiang Province, China) was then added to the sunflower oil (5 g, *w*/*w*), followed by mixing at room temperature for 30 min using magnetic stirring. The two phases were homogenized at room temperature using an Ultra-Turrax T18 homogenizer (ANGNI Co. Ltd., Shanghai, China) at 20,000 rpm according to the manufacturer for 5 min to form a coarse emulsion, followed by high pressure homogenization using an D-6L ultra-high pressure homogenizer (PhD Technology Co., Ltd., Saint Paul, MN, USA) at various pressures. The prepared oil in water (O/W) nanoemulsions comprises by SPI, PC, sunflower oil and β-carotene. The freshly-prepared (O/W) emulsion was used for the following studies.

### 2.3. Measurement of Nanoemulsion Particle Size, Polydispersity Index (PDI) and ζ- Potential

The particle size distribution and PDI for nanoemulsions was determined using dynamic light scattering (Zetasizer Nano-ZS90, Malvern Instrument Co., Ltd., Worcestershire, UK) with a phosphate buffer solution dilution of 1:1000 *v*/*v* using the method of Salvia-Trujillo, L. (2015) [17]. Prior to analysis, the emulsions were diluted to a suitable concentration in phosphate buffer to prevent multiple scattering. The refractive index was taken as 1.46 for emulsion particles and 1.33 for the aqueous dispersion medium as recommended by the manufacturer. Particle size was expressed as the volume weighted mean diameter D_[4,3]_. Definition of the PDI was a dimensionless measure of the broadness of the size distribution calculated from the cumulants analysis. The ζ-potential of the freshly-prepared emulsion was determined using a ζ-potential analyzer (Zetasizer Nano-ZS90, Malvern Instrument Co., Ltd., Worcestershire, UK) with a dilution of 1:105 *v*/*v* with DI [18].

### 2.4. Measurement of Turbidity

The emulsions were diluted 40 times with phosphate buffer solution and phosphate buffer was used as the blank. Absorbance was measured using an LW-1600FC UV spectrophotometer (Shanghai Jinghua Technology Equipment Company, Ltd., Shanghai, China) at 600 nm. Turbidity (T) was calculated as in [19], using the formula:

*T* = 2.302 × (*A* × *V*)/*I* (1)

*A* = Absorbance of diluted emulsion at 600 nm measured against the blank. *V* = Dilution (40). *I* = Optical path length (1 cm).

### 2.5. Turbiscan Stability Index

The stability of the nanoemulsions were measured using the Turbiscan Lab Expert Concentration System Stability Analyzer (France Formulaction Company, Toulouse, France). The measured reflected light is directly related to the particle volume concentration and the average particle size. The instrument mainly uses the central part of the reflected light spot. Reference Luo M et al. (2017), take 18 mL of β-carotene nanoemulsions in Turbiscan special cylindrical glass. Scan every 30 min at 55 °C for 6 h [20]. The Turbiscan Stability Index (TSI) is an emulsion stability index calculated with a Turbiscan lab expert for rapid stability of the emulsion.

### 2.6. Emulsificaton Yield

The β-carotene in the nanoemulsions was extracted with an organic phase of ethanol/*n*-hexane (1:1), and the β-carotene content was analyzed by HPLC. The C30 column was used. The mobile phase was methanol and methyl tert-butyl ether. The flow rate was 1.0 mL/min. The gradient elution was 20 μL. The sample was injected at 450 nm with a DAD detector. The data was processed using Dionex Chromeleon software to determine the content of β-carotene in the SPI-PC nanoemulsions, and the emulsification yield of the SPI-PC nanoemulsions was calculated by the following formula:

(2) $$\mathrm{Emulsification}\;\mathrm{yield}\;\left(\%\right)=\frac{\beta -\mathrm{carotene}\;\mathrm{content}\;\mathrm{in}\;\mathrm{nanoemulsions}}{\mathrm{total}\;\beta -\mathrm{carotene}\;\mathrm{content}}\text{×}100$$

### 2.7. Nanoemulsions Morphology by Confocal Laser Scanning Microscopy

SPI can be stained by Nile Blue dye (1%, *w*/*v*), emitting green fluorescence. Sunflower oil droplets, however, are stained by Nile Red dye (0.1%, *w*/*v*), which do not fluoresce in most polar solvents, but can fluoresce intensely with reddish colors upon placing in a lipid-rich environment. A Leica TCS SP2 confocal laser scanning microscope (Leica Microsystems, Heidelberg GmbH, Heidelberg, Germany) was employed to examine the structure of SPI-PC nanoemulsions. An aliquot of 10 mL of stained nanoemulsions was placed on a microscope slide, and then gently covered with a coverslip. Then, the microstructure of SPI-PC nanoemulsions was determined by confocal laser scanning microscopy.

### 2.8. 3D Raman Imaging of Nanoemulsions Morphology

In this study, the interface structure of the nanoemulsions was analyzed using a confocal Raman imaging system (AIR MP Raman-11, Shimadzu Co., Ltd., Shimane, Japan) with laser excitation at 532 nm and exposure time of 7 s [21]. Droplets were placed on a glass slide and the 5 mW laser focused onto the droplets. Raman scattering signals were focused into the slit entrance of a spectrometer. The scan distances along the X- and Y-axes were adjusted depending on the scan range using the Win Spec 32 software (included with the spectrometer) to automatically collect the spectrum at each point in 2 um increments. The three-dimensional imaging was based on the two-dimensional imaging, while manually adjusting the laser focus depth (i.e., the Z-axis), which the computer then integrated to create the three-dimensional image.

### 2.9. Statistical Analysis

All the experiments were done in triplicate, and the results are presented as mean ± standard deviation. Means were compared using one-way ANOVA followed by Duncan’s test (*p* < 0.05). Statistical analysis was done using SPSS (SPSS Inc., Chicago, IL, USA).

## 3. Results and Discussion

### 3.1. Effect of SPI Addition

The effect of SPI addition on nanoemulsion is shown in Table 1. The particle size and the PDI of nanoemulsions decreased significantly between 1 and 1.5% SPI to its minimum, while the PDI was minimal at 1.5%, suggesting that the smallest, most stable droplets were formed at that concentration. With higher concentrations, both measurements showed larger, less stable particles. Hebishy et al. (2015) studied the effect of protein concentration on the particle size of whey protein-stabilized nanoemulsions, where the emulsions containing 1% protein had the largest droplets, which is consistent with the current results [22]. Li et al. (2014) found that an increase in SPI addition suggested that more protein was available to cover the surface of the droplets, which would lead to smaller droplets and also help prevent aggregation [23]. However, additional protein leads to less stability and larger droplets, due to the presence of excess unabsorbed soluble proteins and the non-protein components leading to depletion flocculation. Emulsification yield followed a similar pattern. However, the turbidity showed a continuous upward trend. The changes above 1.5% SPI could be due to the additional SPI aggregating on the surface, leading to ionic bonding lowering the net charge of the particles, which may lead to aggregation as suggested by the increase in turbidity [24]. Song et al. (2013) suggested that excess protein may form micelles or aggregates with high-pressure homogenization [25]. Berton, Genot & Ropers (2011) found that the unadsorbed proteins increased 2% [26]. Berton et al. (2015) thought that these unadsorbed proteins could also remove proteins from the emulsion surface (depletion flocculation), leading to the physical and chemical destabilization of the emulsion [27].

The ζ-potential was also maximal at 1.5% and 2.0% SPI and above absolute 30 mV. Previous work has suggested that when the absolute value of the ζ-potential is greater than 30 mV, emulsion droplets are stabilized by electrostatic repulsion [15]. The results of Sun et al. (2015) showed that smaller droplets led to an increase in surface energy, which is another measure of the greater stability of smaller particles [28].

### 3.2. Effect of PC Addition

The effect of PC addition levels on the properties of the SPI-PC nanoemulsions is shown in Table 2. The hydrophobic fatty acid groups of the PC can interact on the oil surface and the hydrophilic glycerophosphate groups can align with the water/protein interface to reduce the interfacial tension, helping to stabilize the emulsion [14]. The PC showed a minimum particle size at 0.20% PC and a minimum PDI at 0.22% PC, suggesting the optimum interaction. This confirms that PC could enhance emulsifiability through its own action, as well as interaction with SPI. The absolute value of the ζ-potential gradually increased above 0.17% PC, and all were above absolute 30 mV. Wang B and Martínez K et al. report that PC can enhance the surface negativity of the SPI, leading to increased repulsion between particles [29,30]. In this study, the emulsification yield increased with the addition of PC up to 0.22%. Thus, 0.20% or 0.22% PC seems to be the best amount to use. The results of J. Li et al. (2014) indicate that the interaction between PC and SPI during the preparation of nanoemulsions could increase the compactness of the interfacial emulsion layer [31]. Negatively charged lecithin was also reported in the study of García-Moreno, Horn, and Jacobsen, suggesting that the ζ-potential of casein was shifted to more negative potentials upon the addition of lecithin [32]. At the same time, the decrease of the average particle size of the SPI-PC nanoemulsion and the increase of the absolute value of the ζ-potential enhanced the stability of the emulsion, and the nutrients were better encapsulated within the nanoemulsion.

### 3.3. Effect of Homogenization Pressure

Homogenization can affect the relative adsorption of soy proteins (in particular β-conglycinin and glycinin), leading to different interfacial compositions [33,34]. As shown in Table 3, increasing the homogenization pressure resulted in a significant (*p* < 0.05) optimum for particle size, PDI, turbidity and TSI, which was in agreement with the findings of Tan and Nakajima [7]. As pressure increases, the distribution becomes more uniform. These results suggest that 100 MPa resulted in the best values.

Excessive pressure may lead to droplets cracking. In this study, homogenization pressure was able to significantly influence the properties of emulsions, such as the shear forces and turbulence—both of which are pressure dependent—produced during homogenization being able to affect the particle size and size distribution [35,36]. In this study, the emulsification interface was accompanied by the phenomenon of “lost stabilization” of the nanoemulsion droplets under the condition of high omogenization pressure (140 MPa) output. At this time, the absolute value of the ζ-potential of the nanoemulsion was significantly reduced.

### 3.4. Effect of Homogenization Cycles 

The effect of homogenization times on nanoemulsion is shown in Table 4. The optima for the different parameters were not consistent with a single choice of cycle time. However, from a commercial perspective, the emulsion yield, if still stable, is presumably the most important. Therefore, a set of 4 cycles is suggested, assuming the cost of running each cycle is less than the value of the additional yield. Sadeghpour Galooyak and Dabir found that both the droplet diameter and the PDI improved when the number of cycles was increased from 2 to 4 for nanoemulsions prepared by microfluidization [37]. In practical applications, the proper ratio of homogenization pressure and number of homogenization cycles plays an important role in the economics.

Increasing the number of homogenization cycles, which leads to a longer time for the mechanical action, results in a smaller system size. Decreasing the particle size of particles or small particle aggregates after multiple homogenization cycles resulted in emulsion droplets with greater stability. However, when the number of homogenization cycles reaches 5, the “overprocessed phenomenon” occurred, with new droplets rapidly aggregating, generating a large amount of energy during the homogenization [35]. High-frequency aggregation and extremely high energy density are two of the main causes for the occurrence of “overtreatment”, which is consistent with the current results [38,39].

### 3.5. Confocal Microscopy Imaging

**Figure 1:**
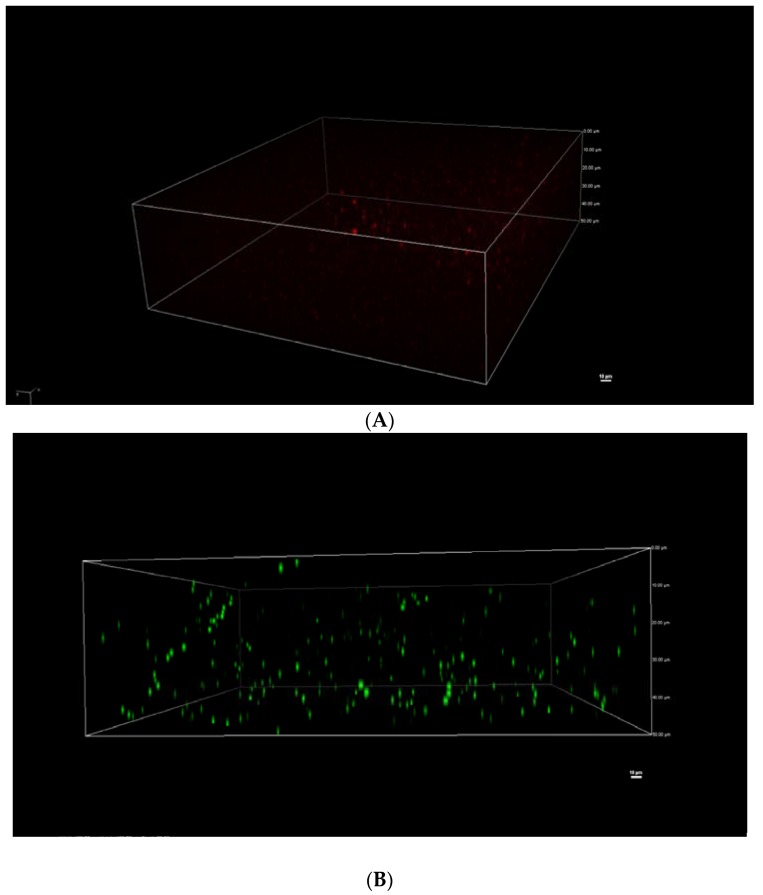
Nanoemulsion morphology using laser confocal microscopy. The red color (**A**) shows the oil droplets, while the green color (**B**) shows the aqueous SPI.

The red fluorescence of Nile red showed the oil phase of the nanoemulsions. It showed that the oil phase was spherical within spherical droplets (Figure 1A). The fluorescence excited by the Nile blue’s green fluorescence (Figure 1B) indicated that the SPI created a continuous and complete coverage of the emulsion surface. It can be seen from Figure 1 that the shape of the nanoemulsions was spherical, indicating that the SPI was completely adsorbed at the interface of the nanoemulsions, the protein of which completely encapsulated the emulsion, showing a core-like structure. Anya Kwan found that orange oil, medium-chain triglyceride (MCT) oil, and Whey Protein Isolate (WPI) could be used to make stable nanoemulsions, and also observed stable spherical nanoemulsions by microscopic confocal microscopy [40].

### 3.6. 3D Raman Imaging

**Figure 2:**
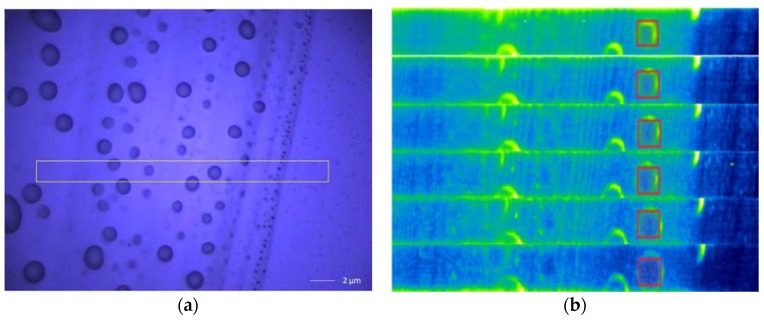
3D-Raman microscopic image of high-pressure homogenous preparation of nanoemulsions. In the figure, (**a**) a single nanoemulsion droplet was selected for analysis; (**b**) 6 Raman imaging plots of the single nanoemulsion droplet were formed as the observational displacement gradually probed into the oil phase.

**Figure 3:**
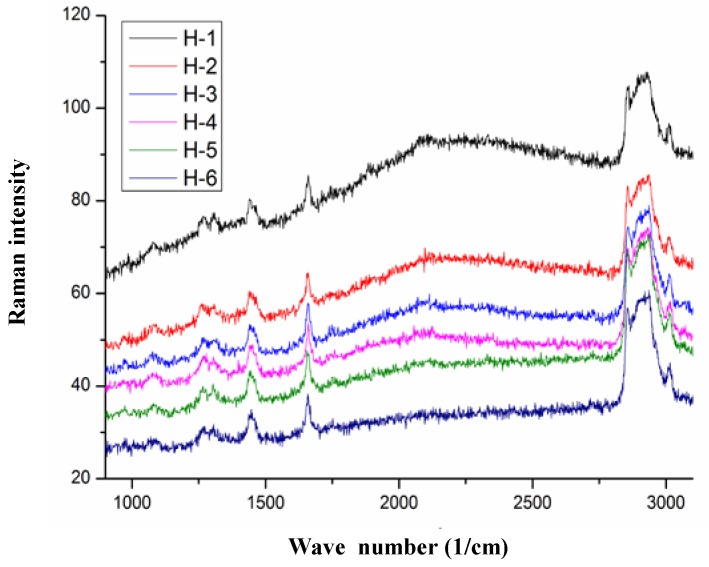
Raman image of high-pressure homogenous preparation of nanoemulsions.

Normally, 3D images help to observe the spatial structural morphology, and the 3D confocal Raman imaging technique is able to simultaneously provide the Raman spectrum results simultaneously [41]. Therefore, in this study, 3D Raman imaging was used to observe the structure of SPI-PC nanoemulsions. A single nanoemulsion droplet was selected for analysis in the yellow box region shown in Figure 2a. As the displacement gradually probed into the center of the oil phase, six Raman imaging formations of the nanoemulsions droplets were formed, as shown in Figure 2b. As the observation gradually explored the center of the oil phase, the green soy protein was more widely distributed at the nanoemulsion interface. It can be seen that, according to our processing conditions, soybean protein can be evenly distributed at the interface of the emulsion droplets. Figure 3 shows characteristic peak of the SPI amide I and the characteristic peaks of the C-H and C-H_2_ groups of PC, which were observed at the peak of 1665, 2840, and 2880 cm^−1^, respectively, further proving that SPI and PC were distributed at the interface of the nanoemulsion droplets. The overall Raman intensity was reduced as the observation angle gradually moved towards the center of oil phase, which was related to the decrease in the distribution of soybean protein in the observed area. However, the characteristic peaks of SPI and PC did not disappear in this process. It was proved that SPI and PC were evenly distributed and interacted at the interface of the nanoemulsion droplets.

## 4. Conclusions

This work was focused on obtaining high-stability SPI-PC nanoemulsions by high-pressure homogenization technology for future applications in the food industry. High-pressure homogenization technology was suitable for producing stabilizing SPI-PC nanoemulsions containing SPI, PC, sunflower oil and β-carotene with good emulsion stability. The results showed that the nanoemulsions had the best stability when SPI was at 1.5%, PC was at 0.22%, the homogenization pressure was 100 MPa, and homogenization was performed 4 times. In addition, confocal microscopy imaging showed that the shape of the nanoemulsions was spherical, with SPI completely adsorbed at the interface of the nanoemulsions, with the protein completely encapsulating the emulsion. Finally, the 3D Raman imaging showed that SPI and PC could be evenly distributed at the interface of the nanoemulsions droplets, according to our processing conditions.

## Figures and Tables

**Table 1:** Effect of soybean protein isolates (SPI) addition on emulsion stability of nanoemulsions.

SPI (%)	Particle Size (nm)	PDI	Zeta Potential (mV)	Emulsificat on Yield (%)	Turbidity	TSI
0.5	735 ± 7 ^a^	0.42 ± 0.03 ^a^	−8.85 ± 0.08 ^d^	80.53 ± 0.80 ^d^	27,000 ± 300 ^f^	5.33 ± 0.05 ^a^
1.0	726 ± 7 ^a^	0.38 ± 0.02 ^b^	−12.73 ± 0.12 ^c^	88.82 ± 0.80 ^b^	28,200 ± 300 ^e^	4.12 ± 0.04 ^c^
1.5	222 ± 2 ^e^	0.26 ± 0.01 ^e^	−33.61 ± 0.33 ^a^	91.25 ± 0.90 ^a^	29,100 ± 300 ^d^	3.03 ± 0.02 ^f^
2.0	395 ± 4 ^d^	0.29 ± 0.00 ^d^	−33.42 ± 0.33 ^a^	90.47 ± 0.90 ^a^	29,900 ± 300 ^c^	3.26 ± 0.03 ^e^
2.5	468 ± 5 ^c^	0.29 ± 0.04 ^d^	−26.81 ± 0.26 ^b^	87.24 ± 0.80 ^c^	34,800 ± 300 ^b^	3.88 ± 0.03 ^d^
3.0	626 ± 6 ^b^	0.31 ± 0.05 ^c^	−15.95 ± 0.15 ^c^	80.79 ± 0.80 ^d^	37,400 ± 300 ^a^	4.34 ± 0.04 ^b^

All samples are average value ± standard deviation (n = 3) and different letters in the same column represent a significant difference between samples (*p* < 0.05). Arrange all the averages in descending order, and use the letter “a” on the maximum average. The amount of phosphatidylcholine addition was 2 mg/mL, the homogenization pressure was 120 MPa, run 4 times.

**Table 2:** Effect of phosphatidylcholine addition on emulsion stability of nanoemulsions.

Phosphatidylch Oline Addition (%)	Particle Size (nm)	PDI	Zeta Potential (mV)	Emulsification Yield (%)	Turbidity	TSI
0.10	433 ± 4 ^a^	0.43 ± 0.05 ^a^	−12.62 ± 0.12 ^d^	84.72 ± 0.84 ^e^	27,300 ± 300 ^d^	5.14 ± 0.05 ^a^
0.17	324 ± 3 ^b^	0.27 ± 0.01 ^c^	−31.03 ± 0.31 ^c^	89.21 ± 0.89 ^c, d^	28,700 ± 300 ^c^	4.41 ± 0.04 ^c^
0.20	222 ± 2 ^c^	0.26 ± 0.02 ^d^	−33.61 ± 0.33 ^b^	91.24 ± 0.91 ^b^	29,200 ± 300 ^a^	3.45 ± 0.03 ^e^
0.22	210 ± 2 ^d^	0.27 ± 0.01 ^c^	−34.75 ± 0.34 ^a^	92.95 ± 0.92 ^a^	29,000 ± 300 ^b^	4.32 ± 0.04 ^d^
0.25	212 ± 2 ^d^	0.29 ± 0.00 ^b^	−34.86 ± 0.34 ^a^	90.12 ± 0.90 ^c^	29,400 ± 300 ^a^	4.76 ± 0.04 ^b^

All samples are average value ± standard deviation (n = 3) and different letters in the same column represent a significant difference between samples (*p* < 0.05). Arrange all the averages in descending order, and use the letter “a” on the maximum average. The amount of soybean protein isolates (SPI) addition was 15 mg/mL, the homogenous pressure was 120 MPa, run 4 times.

**Table 3:** Effect of homogenization pressure on emulsion stability of nanoemulsions.

Homogenization Pressure (MPa)	Particle Size (nm)	PDI	Zeta Potential (mV)	Emulsification Yield (%)	Turbidity	TSI
60	433 ± 4 ^a^	0.43 ± 0.02 ^a^	−18.62 ± 0.18 ^d^	63.21 ± 0.63 ^d^	36,600 ± 400 ^b^	5.72 ± 0.05 ^a^
80	312 ± 3 ^c^	0.37 ± 0.00 ^b^	−31.24 ± 0.31 ^b^	85.63 ± 0.85 ^b^	30,800 ± 300 ^c^	4.94 ± 0.04 ^b^
100	205 ± 2 ^e^	0.20 ± 0.00 ^d^	−31.36 ± 0.31 ^b^	92.57 ± 0.92 ^a^	29,800 ± 300 ^d^	3.66 ± 0.03 ^d^
120	222 ± 2 ^d^	0.26 ± 0.00 ^c^	−33.62 ± 0.33 ^a^	91.24 ± 0.90 ^a^	29,300 ± 300 ^e^	3.41 ± 0.03 ^e^
140	385 ± 3 ^b^	0.45 ± 0.01 ^a^	−20.89 ± 0.20 ^c^	82.83 ± 0.82 ^c^	40,500 ± 400 ^a^	4.78 ± 0.04 ^c^

All samples are average value ± standard deviation (n = 3) and different letters in the same column represent a significant difference between samples (*p* < 0.05). Arrange all the averages in descending order, and use the letter “a” on the maximum average. The amount of soybean protein isolates (SPI) addition was 15 mg/mL, phosphatidylcholine addition was 2 mg/mL, run 4 times.

**Table 4:** Effect of number of homogenizing cycles on emulsion stability of nanoemulsions.

Number of Homogenizing Cycles	Particle Size (nm)	PDI	Zeta Potential (mV)	Emulsification Yield (%)	Turbidity	TSI
2	291 ± 2 ^b^	0.21 ± 0.01 ^c^	−28.23 ± 0.22 ^c^	83.22 ± 0.24 ^e^	29,700 ± 200 ^b^	4.62 ± 0.04 ^c^
3	295 ± 2 ^a^	0.23 ± 0.01 ^b^	−30.84 ± 0.23 ^b^	88.81 ± 0.66 ^c^	29,900 ± 200 ^b^	3.43 ± 0.03 ^e^
4	222 ± 2 ^c^	0.26 ± 0.02 ^a^	−33.66 ± 0.33 ^a^	91.20 ± 0.91 ^a^	29,300 ± 200 ^c^	4.24 ± 0.04 ^d^
5	210 ± 2 ^d^	0.25 ± 0.03 ^a^	−33.98 ± 0.31 ^a^	90.81 ± 0.34 ^b^	29,100 ± 100 ^c^	4.75 ± 0.04 ^b^
6	225 ± 2 ^c^	0.27 ± 0.01 ^a^	−31.21 ± 0.21 ^b^	87.52 ± 0.77 ^d^	30,600 ± 300 ^a^	5.12 ± 0.05 ^a^

All samples are average value ± standard deviation (n = 3) and different letters in the same column represent a significant difference between samples (*p* < 0.05). Arrange all the averages in descending order, and use the letter “a” on the maximum average. The amount of soybean protein isolates (SPI) addition was 15 mg/mL, phosphatidylcholine addition was 2 mg/mL and the homogenization pressure was 120 MPa.
